# *Aequatobolbus*, a new South American genus of Bolboceratinae (Coleoptera: Scarabaeoidea: Bolboceratidae) from Ecuador

**DOI:** 10.7717/peerj.21107

**Published:** 2026-04-10

**Authors:** Julián Clavijo-Bustos, Josué Franco-Salgado

**Affiliations:** 1 Sección de Entomología, Colecciones Biológicas, Centro de Colecciones y Gestión de Especies, Instituto de Investigación de Recursos Biológicos Alexander von Humboldt, Villa de Leyva, Boyacá, Colombia; 2 Instituto Nacional de Biodiversidad, Quito, Ecuador; 3 Laboratorio de entomología del museo QCAZ, Pontificia Universidad Católica del Ecuador, Quito, Ecuador

**Keywords:** Neotropics, Taxonomy, Identification key, Distribution, Scarabs

## Abstract

*Aequatobolbus otongachi*, **gen. et sp. nov.**, the new South American genus and species of Bolboceratinae, are described from Ecuador. The new genus and species are diagnosed and illustrated, and an updated key to the South American genera of Bolboceratinae is given. This is the ninth genus of Bolboceratinae from South America and one of the most northern distributed. It is morphologically related to *Bolbapium* Boucomont, 1910 and *Parabolbapium* Ide & Martínez, 1994, but it is easily distinguished from both by its longer ocular canthus.

## Introduction

Bolboceratinae is one of the two subfamilies of Bolboceratidae Mulsant, 1842 (Coleoptera: Scarabaeoidea) (*sensu* Scholtz & Grebennikov, 2016) and, with approximately 36 genera and 300 species distributed worldwide, it is the richest subfamily within the family (Scholtz & Grebennikov, 2016). In South America, the subfamily exhibits an almost entirely unique taxonomic composition, with most genera and species being nearly exclusively distributed in the subcontinent (Mondaca & Smith, 2008; Carvalho & Vaz-de Mello, 2022; Dantas, Ferreira & Bravo, 2024; Clavijo-Bustos, 2024; Boilly & Vaz-de Mello, 2025). Currently, eight genera and 44 species of Bolboceratinae are known from South America (Martínez, 1976; Ide & Martínez, 1993; Ide & Martínez, 1994; Mondaca & Smith, 2008; Carvalho & Vaz-de Mello, 2022; Dantas, Ferreira & Bravo, 2024; Boilly & Vaz-de Mello, 2025), but additional taxa are presently being described by us. This suggests that there are still species awaiting discovery and formal description in the subcontinent. It is also noteworthy that, contrary to the most common latitudinal diversity pattern, the number of Bolboceratinae species and genera in the Americas decreases toward the Equator and increases toward subtropical and temperate zones.

During the last 60 years, most generic reclassifications of the South American Bolboceratinae species have taken place, assigning them to new or existing genera (*e.g*., Howden, 1973; Martínez, 1976; Mondaca & Smith, 2008). More recently, over the last two decades, the richest genera have undergone taxonomic revision, resulting in the description of several new species (Mondaca & Smith, 2008; Carvalho & Vaz-de Mello, 2022; Clavijo-Bustos, 2024; Boilly & Vaz-de Mello, 2025; J Clavijo-Bustos, 2025, unpublished data). The discovery in an Ecuadorian entomological collection of five female specimens belonging to an undescribed genus and species led us to the present article, which describes *Aequatobolbus otongachi* **gen. et sp. nov.** The new genus and species inhabit montane cloud forest of the Ecuadorian Chocó Biogeographic Region (Fagua & Ramsey, 2019), and the closest morphologically related genera are *Bolbapium* Boucomont, 1910 and *Parabolbapium* Ide & Martínez, 1994.

## Materials & Methods

The revision of the specimens was conducted under an Olympus SZ61 stereomicroscope. Presentation of type specimen labels follows: a vertical bar “ — ” is used to separate lines within a label, two slashes “ // ” denote different labels, and label characteristics are enclosed in square brackets “[ ]”.

Type specimens of the new genus were collected by Dr. Giovanni Onore using a Malaise trap in the Otongachi Protected Area (Pichincha Province) in 2005 and 2021. The specimens were deposited by the collector in the institution listed below, where they were located by the authors during an academic visit in 2025; the housing institution subsequently provided them on loan to the authors for examination:

QCAZ-I División de Invertebrados, Museo de Zoología de la Pontificia Universidad Católica del Ecuador, Quito, Ecuador (DJ Vela Peralta, MF Salazar Buenano, F Maza).

The specimens were softened by submersion into boiled water, then, the sclerotized internal structures were dissected and placed into a glycerin-filled vial below the respective specimen on the same pin (female terminalia and mouthparts) or, in the case of hindwing, it was extended and glued onto an acid-free card.

Terminology for external morphology follows Mondaca & Smith (2008), Lawrence et al. (2010) and Carvalho & Vaz-de Mello (2022); for female terminalia, Cristóvão & Vaz-de-Mello (2021); for the hindwing, Lawrence et al. (2021); and for the mouthparts, Nel & De Villiers (1988) and Nel & Scholtz (1990).

The photographs were taken with a Canon^®^ EOS Rebel T7i coupled to a StackShot^®^ extended macro rail package with Canon^®^ EF 100 mm f/2.8 macro lens and Venus Laowa^®^ 25 mm F/2.8 Ultra-Macro lens. Focus stacking was performed using Helicon Focus *v.* 7.5.6 (Helicon Soft Ltd.) and the images were edited using Adobe Photoshop Lightroom Classic (*v.* 12. 4) and Adobe Photoshop CS6. The measurements were taken with IC Measure 3.0.0.521. The type locality was mapped using QGIS *v.* 3.38.3-Grenoble (QGIS.org 2025).

The electronic version of this article in Portable Document Format (PDF) will represent a published work according to the International Commission on Zoological Nomenclature (ICZN), and hence the new names contained in the electronic version are effectively published under that Code from the electronic edition alone. This published work and the nomenclatural acts it contains have been registered in ZooBank, the online registration system for the ICZN. The ZooBank LSIDs (Life Science Identifiers) can be resolved and the associated information viewed through any standard web browser by appending the LSID to the prefix http://zoobank.org/. The LSID for this publication is: urn:lsid:zoobank.org:pub:EBF3C6AF-6FB8-419D-8F2D-014E90B970E9. The online version of this work is archived and available from the following digital repositories: PeerJ, PubMed Central SCIE and CLOCKSS.

## Results

**Table:** 

**Figure 1:**
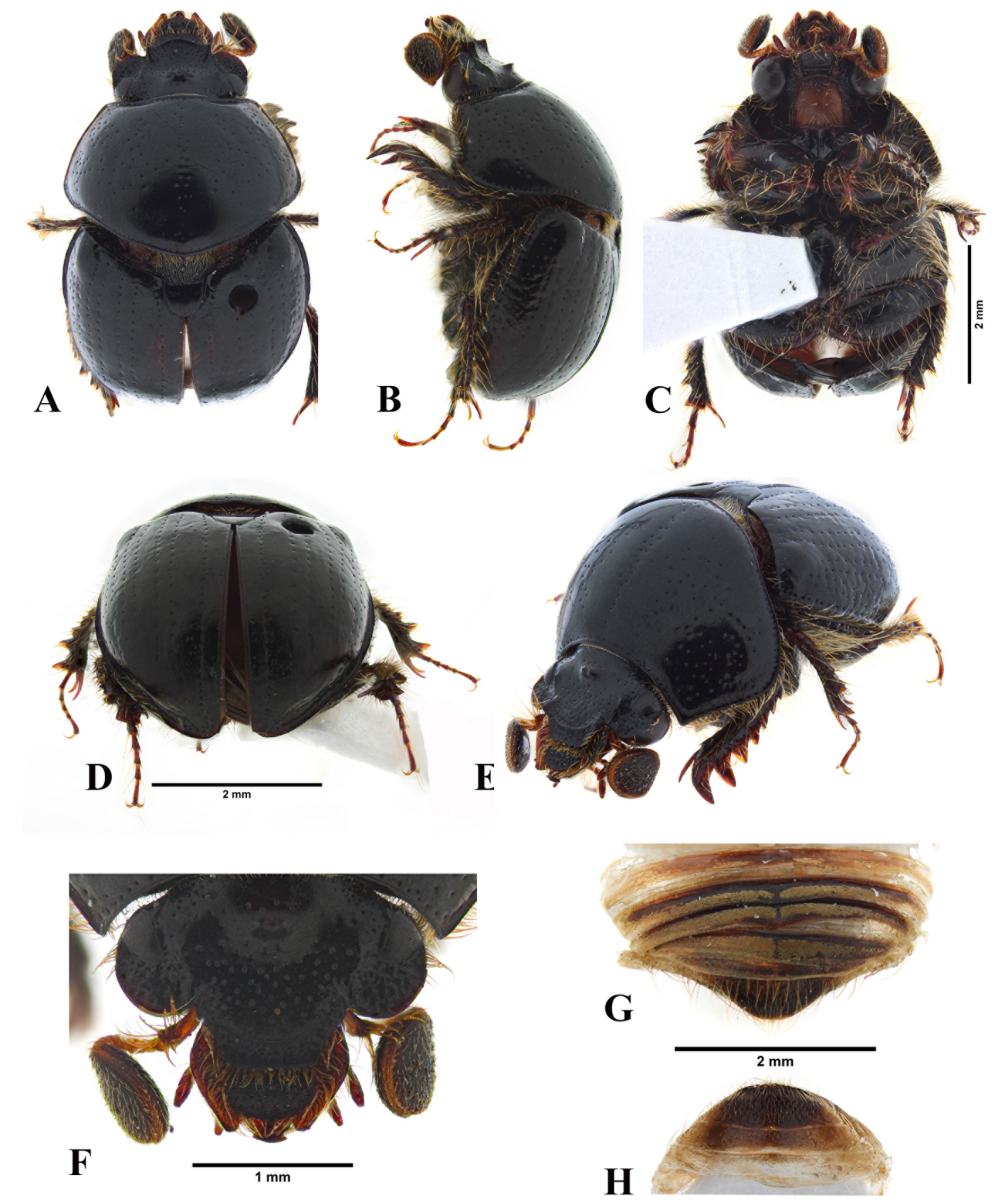
*Aequatobolbus otongachi* gen. et sp. nov., female holotype. (A) Habitus in dorsal view. (B) Habitus in lateral view. (C) Habitus in ventral view. (D) Habitus in posterior view. (E) Habitus in antero-dorsal view. (F) Head in dorsal view. (G) Abdomen in dorsal view. (H) Abdomen in ventral view.

**Figure 2:**
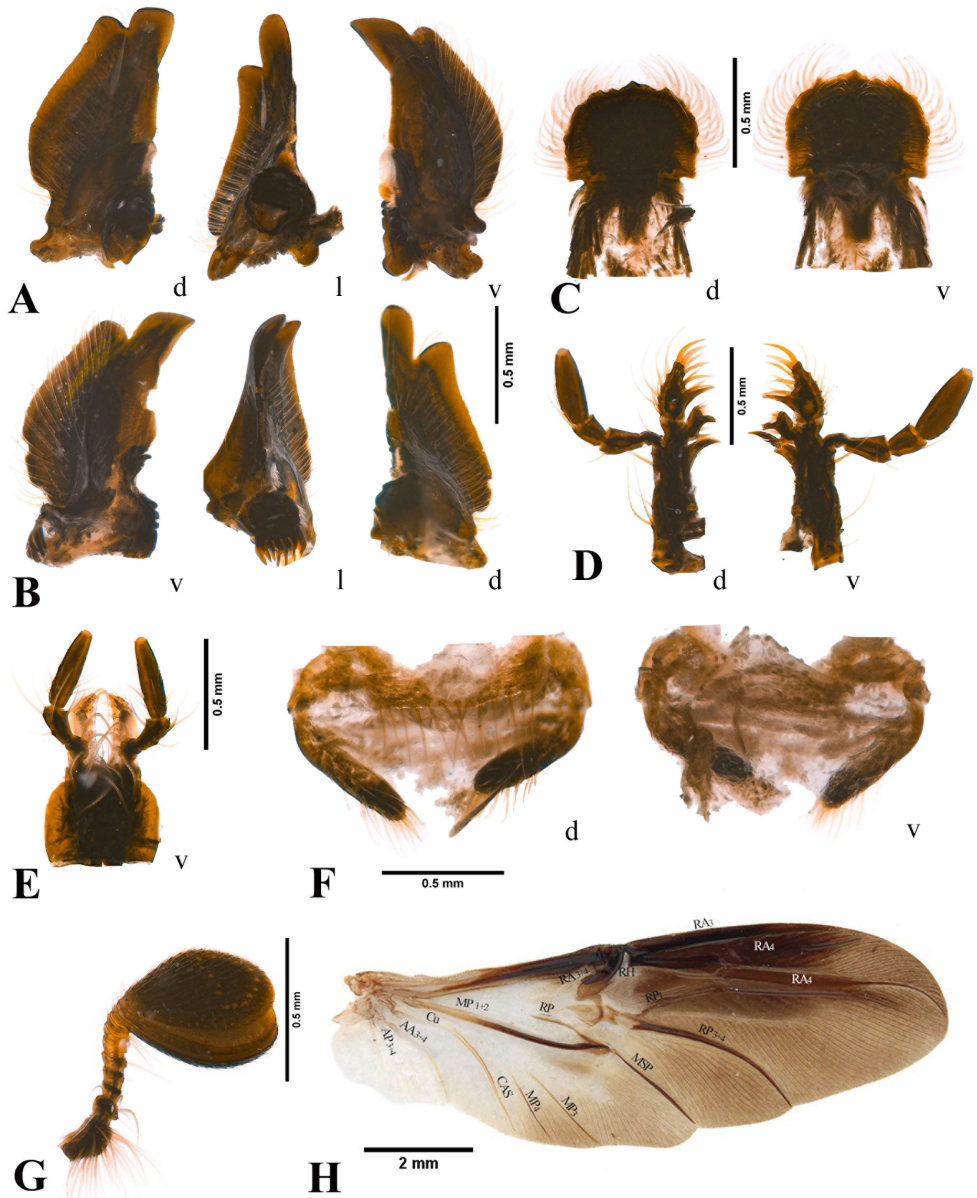
*Aequatobolbus otongachi* gen. et sp. nov., female paratype. (A) Left mandible. (B) Right mandible. (C) Labrum. (D) Left maxilla. (E) Labium. (F) Female genitalia. (G) Right antenna. (H) Left hindwing in dorsal view. d, dorsal view; l, lateral internal view; v, ventral view.

**Figure 3:**
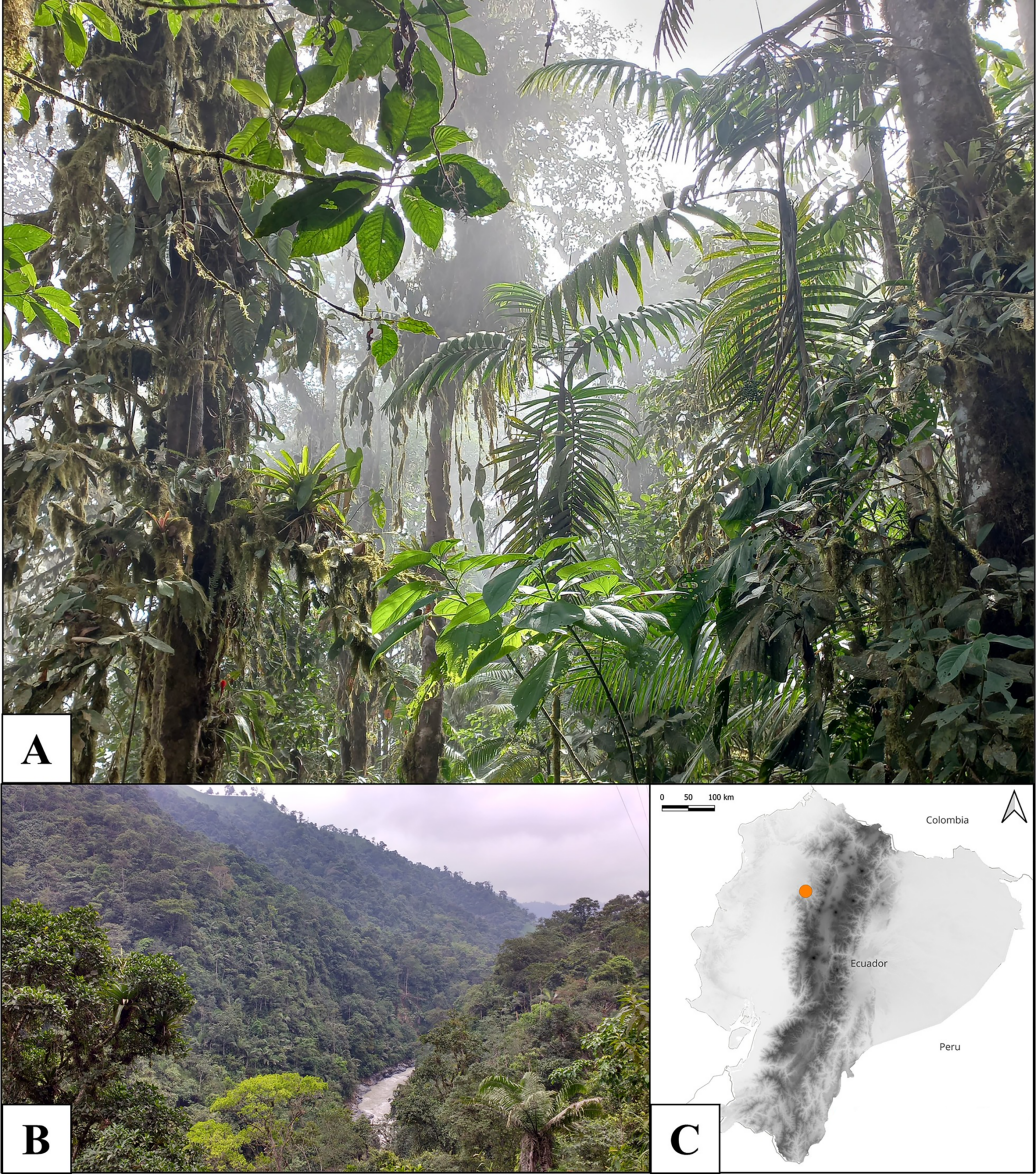
*Aequatobolbus otongachi* gen. et sp. nov. (A) Habitat of type locality. (B) Landscape of type locality. (C) Distribution map. Photo credit: W Pruna and G Onore.

**Figure 4:**
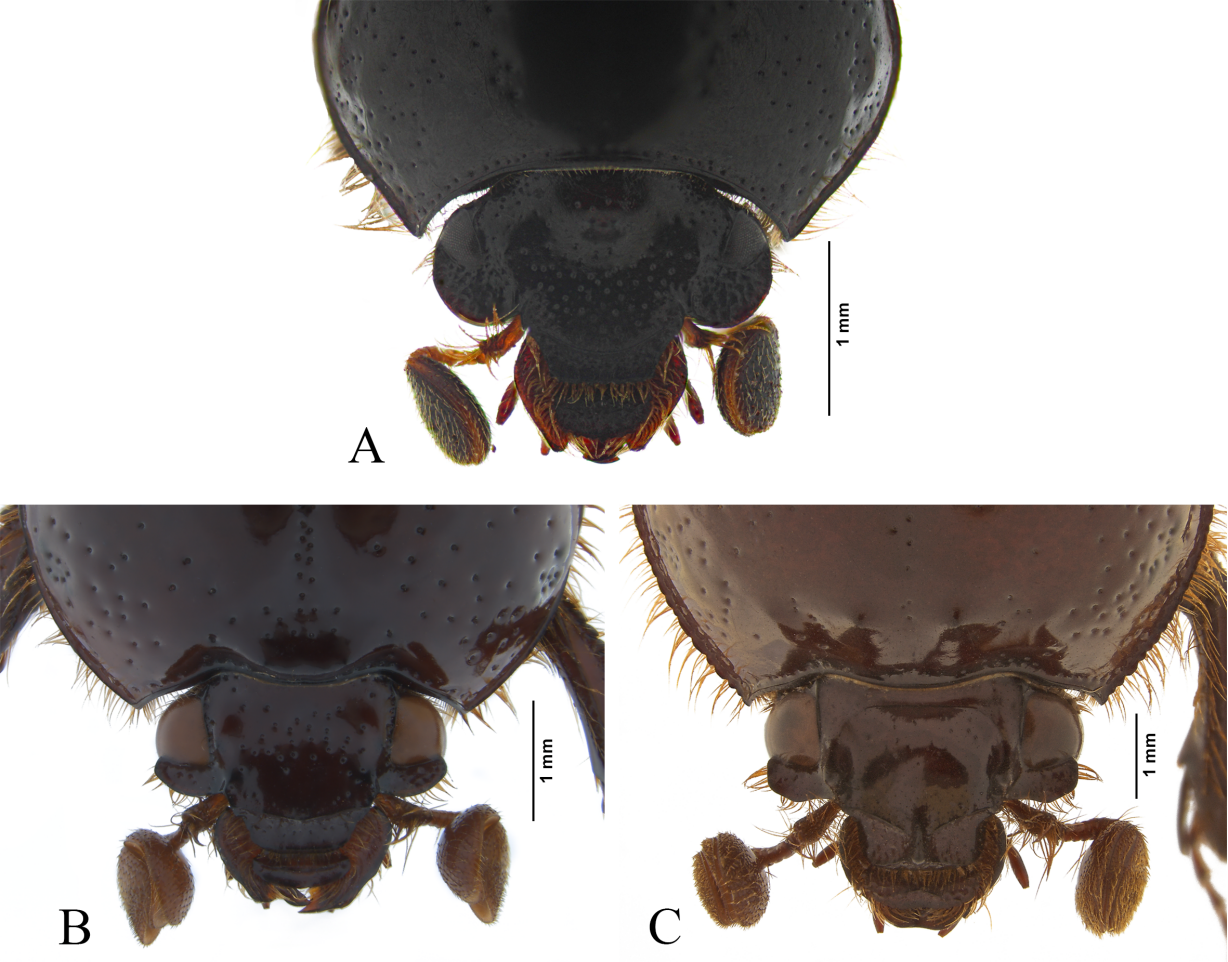
Head of some South American genera of Bolboceratinae. (A) *Aequatobolbus otongachi* gen. et sp. nov. (B) *Bolbapium caesum* (Klug, 1843). (C) *Parabolbapium* sp.

Taxonomy
Superfamily Scarabaeoidea Latreille, 1802
Family Bolboceratidae Mulsant, 1842
Subfamily Bolboceratinae Mulsant, 1842
***Aequatobolbus*** ** Clavijo-Bustos & Franco, new genus**
(Figs. 1–3, 4A)
urn:lsid:zoobank.org:act:28A2E642-9D0E-41A4-B977-773215A34715

**Type Species.** *Aequatobolbus otongachi*, new species, here designated.

**Diagnosis.** Total length 5.40–5.80 mm. Body shape spherical, strongly convex; color uniformly black, surface shiny. Clypeus trapezoidal; frons in the middle with two small, acute tubercles; scutellar shield subpentagonal, smooth; elytra with five striae between suture and humeral callus; mesocoxae evidently separated. Female genitalia with gonocoxites long narrow, with apex rounded and setose.

**Description** (Fig. 1)**.** Total length 5.40–5.80 mm. Body shape spherical, strongly convex. Uniformly black, surface shiny.

Head slightly trapezoidal, narrow apically (Figs. 1F, 4A). Vertex smooth, laterally with small rounded punctures densely distributed (Figs. 1F, 4A). Frons in the middle with two small, acute tubercles; also, one interocular tubercle present over the base of each ocular canthus, each tubercle smaller than medial tubercles; surface with scattered small rounded punctures around the medial tubercles, anteriorly densely distributed (Figs. 1F, 4A). Canthus partially divides the eye, longer than the dorsal portion of the eye, anterior edge widely rounded, apex acute; surface with small rounded punctures densely distributed (Figs. 1F, 4A). Frontoclypeal suture evenly rounded, laterally ending in an acute small tubercle (Figs. 1F, 4A). Clypeus subtrapezoidal; surface with moderately coarse, small rounded punctures giving a subrugose appearance (Figs. 1F, 4A). Antennae with 11 antennomeres, the last three globose and built into an ovoid club; scape and pedicel setose, with long pale setae (Fig. 2G).

Mandibles and labrum exposed beyond clypeus in dorsal view (Figs. 1F, 4A). Mandibles asymmetric, strongly inward curved, one tooth present in the scissorial area of the apicalis, prostheca present, and molar area of the basalis with a single molar lobe subrounded in inner lateral view; molar lobes of each mandible asymmetric, the surface is slightly concave in the left mandible and convex in the right; outer edge of mandibles with a notch before of the scissorial area resulting in a rounded lobe; tooth of scissorial area with rounded apex in left mandible and acute apex in right mandible (Figs. 2A–2B). Labrum quadrangular, with the anterior edge of the distal epipharynx widely rounded, with a small median tooth, but lacking corypha; edges of the distal epipharynx setose; proximal epipharynx with microsensilla in the central posterior zone (Fig. 2C). Maxillae with the cardo projected horizontally, basistipes long; galea and lacinia clearly separated, each with a boot-like shape; galea divided, distagalea setose and larger than proxagalea, and with a median acute projection basally; lacinia apically sclerotized, long, reaching the base of the distagalea, maxadentes and proxadentes present; maxillary palps composed of four palpomeres (Fig. 2D). Labium with submentum and mentum separated, and mentum and prementum fused; submentum with border of anterior third setose; mentum quadrangular, anteriorly with few long setae in the middle; prementum with two labial palps composed of three palpomeres; prementum also with two ligular lobes of long rectangular shape and setose, which are extended beyond the prementum (Fig. 2E).

Pronotum transverse, wider than long, convex; anterior angles right-angled, posterior angles widely rounded; base widely projected posteriorly in the middle (Figs. 1A, 1E). Margins with a row of small rounded punctures distanced by 1–3 times one puncture diameter; disc of pronotum with a longitudinal medial shallow sulcus and its surface with an irregular row of small rounded punctures sparsely distributed, also, punctures sparsely distributed laterally to medial sulcus, denser over lateral sides (Figs. 1A, 1E).

Ventrally, hypomeron surface densely setose, with long pale setae (Fig. 1C). Prosternum extremely short compared with mesosternum, with the surface setose (Fig. 1C). Mesosternum wider than long (Fig. 1C). Metaventrite convex, with a longitudinal medial narrow sulcus; surface sparsely setose, except over the central area of the disc and more densely setose over the lateral sides of the lateral arms of the disc (Fig. 1C).

Scutellar shield as long as wide, subpentagonal; surface smooth, shiny (Fig. 1A).

Elytron longer than wide, convex (Figs. 1A, 1D). Disc with five shallowly impressed striae between suture and humeral callus, 10 in total; each elytral striae with a longitudinal row of small rounded punctures distanced by 3–5 times one puncture diameter, closer in the first stria; humeral callus bulbous, elytral apical callus not evident (Figs. 1A, 1D). Elytral intervals smooth (Figs. 1A, 1D). Epipleura gradually narrows toward apex.

Hindwing lacking anal embayment; vein AP3+4 short, vein AA3+4 present, curved and parallel to the CAS; veins MP3 and MP4 free individually, parallel to each other; vein RP3+4 apically curved; veins RA3 and RA4 after radial hinge (RH), continuing separately but both strongly sclerotized (Fig. 2H).

Legs with procoxae contiguous, mesocoxae widely separated, and metacoxae contiguous (Fig. 1C). Ventral surface of profemur sparsely setose. Protibia with posterior edge dentate, apical tooth larger than the previous and the remaining teeth decreasing in size toward base; dorsal surface with a median row of setae; protibial spur apically acute and slightly downward curved, slightly shorter than the combined length of the protarsomeres I–III; protarsomeres I and V subequal in length and each shorter than the combined length of the protarsomeres II–IV, protarsomeres II–IV gradually decreasing in length (Figs. 1A–1E). Ventral surface of mesofemur smooth, setose over the anterior and posterior borders (Fig. 1C). Mesotibia shorter than mesofemur, curved; posterior surface sparsely setose in the base; anterior surface dentate, teeth increasing in size apically; ventral surface with a row of setae; mesotibial spurs straight, dorsal spur longer than ventral spur and subequal to the length of the mesotarsomere I; mesotarsomeres I and V subequal in length and each shorter than the combined length of the mesotarsomeres II–IV, mesotarsomeres II–IV gradually decreasing in length (Figs. 1A–1E). Ventral surface of metafemur smooth, setose over the anterior and posterior borders (Fig. 1C). Metatibia as long as metafemur, less curved than mesotibia, otherwise like mesotibia; metatarsomeres like mesotarsomeres; metatibial spurs straight, dorsal spur longer than both ventral spur and metatarsomere I (Figs. 1A–1E).

Abdomen largely hidden by metathoracic legs and elytra. Abdominal ventrites slightly convex in the middle, the last two evidently sclerotized (Fig. 1H). Abdominal ventrites II–V moderately setose laterally, with few short pale setae, which are longer at the lateral borders; abdominal ventrite VI slightly longer than previous, apex truncate, with long pale setae (Fig. 1H). Pygidium subtriangular, apex rounded, surface with sparse long setae (Fig. 1G).

Female terminalia with dorsal paraproct as one wide plate, apically with an irregular row of setae; lateral paraprocts apically rounded and setose (Fig. 2F). Gonocoxites long narrow, with apex rounded and setose (Fig. 2F).

**Etymology.** The generic name is composed of the word *aequator*, the obsolete Latin form of *equator*, in reference to the geographical line at latitude 0° and the country Ecuador; and the suffix—*bolbus*, commonly used for many Bolboceratinae genera, from the Greek *bolbos* meaning ‘bulb-shaped’, referring to the strongly convex body form of members of the subfamily. The genus name is masculine.

**Geographic distribution.** Known only from Ecuador, in the northwestern part of Pichincha Province. *Aequatobolbus* **gen. nov.** was recorded in Otongachi Protected Area, a montane cloud forest remnant of approximately 150 ha (Fig. 3).

**Taxonomic remarks.** *Aequatobolbus* **gen. nov.** is most similar to *Bolbapium* and *Parabolbapium*, sharing the shape of antenna, the subpentagonal shape of the scutellar shield, five striae between elytral suture and humeral callus, and mesocoxae evidently separated (Martínez, 1976; Ide & Martínez, 1994). In addition, the trapezoidal shape of clypeus is also shared with *Bolbapium*, not having it subdivided into three subtriangular regions by two oblique carinae as occurs in *Parabolbapium*.

*Aequatobolbus* can be distinguished from *Bolbapium* and *Parabolbapium* externally by its black color (Figs. 1, 4A) (dark brown to reddish brown in the other two genera, Figs. 4B–4C); long ocular canthus, which is longer than the dorsal portion of the eye (Figs. 1F, 4A) (shorter in the other two, Figs. 4B–4C); the shallowly impressed elytral striae (Fig. 1A, 1D) (deeply impressed in the other two). Internally, the mouthparts of *Aequatobolbus* are very different from those of *Bolbapium* and *Parabolbapium*: mandibles are less asymmetric, both left and right mandible have a notch before of the scissorial area resulting in a rounded lobe, and the scissorial area of both mandibles has a single apical tooth (Figs. 2A–2B) (notch present just in right mandible, and apex of scissorial area of both mandibles with two teeth in the other two genera); the distagalea of maxilla with a median acute projection basally (Fig. 2D) (not present in the other two); the widely and sinuous shape of maxadentes (Fig. 2D) (narrow laminar in the other two); labrum quadrangular, the distal epipharynx with the anterior edge widely rounded and the lateral edges complete (Fig. 2C) (labrum transversal rectangular, anterior edge truncate and emarginate medially, and lateral edges with a strong median indentation in the other two). Also, the hindwing of *Aequatobolbus* differs from the other two genera in: lacking anal embayment; vein AA3+4 curved and parallel to the CAS; vein RP3+4 apically curved; and veins RA3 and RA4 after radial hinge (RH), strongly sclerotized and hard to distinguish between them (Fig. 2H).

From other South American genera, *Aequatobolbus* is distinguished by the characters mentioned in the key for the genera identification presented at the end of this article.

**Table:** 

***Aequatobolbus otongachi*** ** Clavijo-Bustos & Franco, new species**
(Figs. 1–3, 4A)
urn:lsid:zoobank.org:act:4B3006A3-C977-4430-A748-66478E98F4C9

**Type material. Holotype**, female: “ECUADOR Pichincha — Otongachi 15JAN2021 —−0.321207. −78.950988 — 850 m Giovanni Onore” [white label, printed] // “HOLOTYPE —*Aequatobolbus otongachi* — gen. et sp. nov — Clavijo-Bustos & Franco, 2026” [red label, printed] // “QCAZ I — 280492” [white label, printed]. **Paratypes.** Four females: 2 *idem*, except for “QCAZ I — 280493” and “QCAZ I — 280495”; 2 *idem*, except for “... 26MAY2005 …”, and “QCAZ I — 280491” and “QCAZ I — 280494”.

Additionally, anecdotal collection data provided by Dr. Giovanni Onore indicates that the specimens were collected using a Malaise trap.

**Description and diagnosis.** Holotype female (Fig. 1): Total length 5.68 mm, maximum width of pronotum 3.39 mm, width of elytral base 3.07 mm. Other aspects of the diagnosis and description of *Aequatobolbus otongachi* are redundant with that of the genus.

**Sexual dimorphism.** All known specimens of the new species are females; the male is unknown. However, closely related South American genera do not exhibit evident sexual dimorphism in external morphology, suggesting the same may apply to *Aequatobolbus otongachi*.

**Variation.** Total length 5.40–5.80 mm, maximum width of pronotum 3.14–3.39 mm, width of elytral base 3.02–3.30 mm.

**Geographic distribution.** Ecuador, province of Pichincha, exclusively known from the type locality, the Otongachi Protected Area (Fig. 3).

**Habitat remarks.** *Aequatobolbus otongachi* **gen. et sp. nov.** was collected in one of the most important biodiversity hotspots in the world, characterized by high levels of endemism (Myers et al., 2000), but also highly threatened. The Chocó Biogeographic Region still preserves significant tracts of original forest and is the second largest expanse of tropical rainforest in South America, after the Amazon Basin. Currently, the rapid and gradual replacement of forested areas by secondary vegetation or urbanization threatens biodiversity, particularly in northern Ecuador (Fagua & Ramsey, 2019). This new discovery highlights the need to strengthen conservation efforts and the importance of preserving forest remnants in endangered regions.

**Etymology.** The epithet refers to the name of the Otongachi Protected Area. The specific name is intended to acknowledge the conservation efforts of the Otonga Foundation and the Otongachi Protected Area. The epithet is a noun in apposition.

### Updated key to the South American genera of Bolboceratinae

(Adapted from Martínez, 1976; Ide & Martínez, 1994; Mondaca & Smith, 2008)

1.: Scutellar shield twice as long as wide, nearly parallel sided ………… …………………………………………………*Bolbothyreus* Howden
-: Scutellar shield as long as wide or nearly, subtriangular to subpentagonal ………………………………………………………………………2
2.: Elytra with five striae between suture and humeral callus ……………3
-: Elytra with seven striae between suture and humeral callus ……………6
3.: Mesocoxae subcontiguous to contiguous, space between them shorter than the length of coxa …………………*Bolbelasmus* (*Eucanthus*) Westwood
-: Mesocoxae widely separated, space between them nearly equal to the length of coxa ………………………………………………………………4
4.: Clypeus subtrapezoidal, divided into three triangular subregions by two oblique carinae extending from median tubercle toward anterior angles; frons strongly produced anteriorly …*Parabolbapium* Ide & Martínez
-: Clypeus subtrapezoidal, not divided into subregions, lacking oblique carinae; frons slightly or not produced anteriorly ……………………………5
5.: Ocular canthus short, shorter than the length of the dorsal portion of the eye ……………………………………………………*Bolbapium* Boucomont
-: Ocular canthus long, as long as the length of the dorsal portion of the eye ……………………………………………*Aequatobolbus* gen. nov.
6.: Mesocoxae widely separated, space between them nearly equal to the length of coxa …………………………………*Halffterobolbus* Martínez
-: Mesocoxae subcontiguous to contiguous, space between them shorter than the length of coxa ……………………………………………………7
7.: Prosternal process prominent; protibial spur extending to apex of protibia ……………………………………………*Bolborhinum* Boucomont
-: Prosternal process greatly reduced to absent; protibial spur not extending to apex of protibia ……………………………………………………8
8.: Mesocoxae subcontiguous, slightly separated; scutellar shield with coarse, dense punctuation; species with sexual dimorphism in head and pronotum ……………………………………………………*Zefevazia* Martínez
-: Mesocoxae contiguous, adjacent one to the other; scutellar shield with shallow, sparse punctuation; species without sexual dimorphism ………… ……………………………………………………*Pereirabolbus* Martínez

## Discussion

The description of *Aequatobolbus otongachi* **gen. et sp. nov.** raises the number of Bolboceratinae genera and species known from South America to nine and 45, respectively. Nevertheless, additional undescribed taxa likely remain, some of which are currently in preparation (Clavijo-Bustos, 2024; J Clavijo-Bustos, 2025, unpublished data). Continued collecting efforts and the examination of previously underrepresented museum collections may further expand known distributions and even reveal additional taxa (Carvalho & Vaz-de Mello, 2022; Clavijo-Bustos, Guillén & Barria, 2022; Boilly & Vaz-de Mello, 2025).

Members of South American Bolboceratinae are widely distributed across the continent, but none had been recorded from the Chocó Biogeographic region prior to this study. The discovery of *Aequatobolbus otongachi* **gen. et sp. nov.** in the cloud forests of this region suggests that Bolboceratinae may also occur in previously unrecorded areas. This also makes *Aequatobolbus* **gen. nov.** one of the northernmost distributed genera of Bolboceratinae in South America.

Notably, the other two genera recorded from northern South America, *Bolbapium* and *Parabolbapium*, appear to be the more closely related to *Aequatobolbus* **gen. nov.** These genera share similarities in external morphology but they can be distinguished from the new genus by the length of the canthus. While *Aequatobolbus* **gen. nov.** and *Bolbapium* share a similar shape of clypeus, hindwing and mouthparts morphology suggest a closer relation between *Bolbapium* and *Parabolbapium* than between either of them and *Aequatobolbus* **gen. nov**.

## Conclusions

A new genus and species of Bolboceratinae from Ecuador is described based on a comprehensive examination of museum specimens. As result, *Aequatobolbus otongachi* **gen. et sp. nov.** represents the ninth genus of the subfamily from South America and is closely related to *Bolbapium* and *Parabolbapium*. This is the first genus and species of Bolboceratinae known from the montane cloud forest of the Chocó Biogeographic Region. Further studies of South American Bolboceratinae, incorporating museum material and expanded collections, may reveal additional undescribed taxa.

##  Supplemental Information

10.7717/peerj.21107/supp-1 Supplemental Information 1 Aequatobolbus otongachi Raw data of occurrences in Darwin Core (DwC).

## Additional Information and Declarations

### Data Availability

The following information was supplied regarding data availability: The raw data is available in the Supplemental File.

### New Species Registration

The following information was supplied regarding the registration of a newly described species: Publication LSID: urn:lsid:zoobank.org:pub:EBF3C6AF-6FB8-419D-8F2D-014E90B970E9 *Aequatobolbus* LSID: urn:lsid:zoobank.org:act:28A2E642-9D0E-41A4-B977-773215A34715 *Aequatobolbus otongachi* LSID: urn:lsid:zoobank.org:act:4B3006A3-C977-4430-A748-66478E98F4C9.
